# Serum SAA1 and APOE are novel indicators for human cytomegalovirus infection

**DOI:** 10.1038/s41598-017-13591-x

**Published:** 2017-10-17

**Authors:** Ni Xie, Zhonghang Li, Ran Zuo, Suwen Qi, Ting Zhu, Litao Liu, Lili Wan, Jianhui Yuan

**Affiliations:** 1 Institute of Translation Medicine, Shenzhen Second People’s Hospital, Sungang West Road, Shenzhen, 518000 China; 2 College of Life Sciences, Shenzhen University, Nanhai Ave 3688, Shenzhen, 518060 China; 3 Institute of Toxicology, Shenzhen Center for Disease Control and Prevention, No 8 Longyuan Road, Nanshan District, Shenzhen, 518055 China

## Abstract

Human cytomegalovirus (HCMV) infection is a global concern and highly infectious. HCMV-infected individuals are often carriers with damaged immunity. However few diagnostic indicators block HMCV control and prevention. Thus, we measured 21 serum proteins related to HCMV infection using iTRAQ-labeling based quantitative proteomic approaches and SAA1 and APOE were confirmed as candidate serum indicators for identification of HMCV infection according to ROC curve analysis and that co-occurrence of SAA1 and APOE are better markers than individual proteins.

## Introduction

Human cytomegalovirus (HCMV) is also known as human herpesvirus-5 (HHV-5) and is within the Betaherpesvirinae subfamily which includes cytomegaloviruses from other mammals^1^. Most HCMV-infected individuals are asymptomatic with latent infection. Once infected, individuals become long-term viral carriers. HCMV is identified in blood mononuclear cells, and some viruses are found in T cells^2^. HCMV infection is typically unnoticed in healthy people, but can be lethal for immunocompromised persons, such as HIV-infected individuals, organ transplant recipients, or newborns. HCMV infection is global and highly infectious; most people acquire the infection during childhood. HCMV can infect NK cells individually and can mimic infectious mononucleosis or glandular fever^3^. Although the mechanisms of HCMV persistence remain unclear, frequent virus shedding into saliva and the genitourinary tract may account for most world-wide infection. The disease associated with HCMV infection is mostly attributed to lytic viral replication and organic damage either secondary to virus-induced cell death or from host immunological responses^4^. Virus-controlled phosphorylation accounts for ganciclovir-resistance and a recently discovered protein kinase homologue may be a potential therapy for HCMV infections^5^.

The chemokine, vCXCL-1s, is key to viral dissemination by binding to different chemokine receptors^6^. Another group reported that microRNA miR-21 can attenuate HCMV replication in neural cells by inhibiting expression of Cdc25a^7^. Studies also indicate that HCMV can evaded the immune system by expressing class I MHC homologue on the infected host cell surface^8^.

Because no therapy exists, early diagnosis is necessary but few biomarkers are available. Here we report that SAA1 and APOE were differentially expressed in HCMV-infected patients and that both proteins were more robust for identifying infection than either protein alone.

## Materials and Methods

### Reagents

A Pierce C18 Spin Columns was purchased from Thermo Scientific (Rockford, IL). ProteoExtract kit was purchased from MERK (Kenilworth, NJ). The iTRAQ 8 Plex kit was purchased from SCIEX (Framingham, MA). The antibodies against GAPDH, Serum amyloid A-1 protein (SAA1) and apolipoprotein E (APOE) were purchased from Abcam (Cambridge, MA). The secondary antibodies and electro-generated chemiluminescence substrates were from Pierce (Rockford, IL). PhosphoSafe Extraction Reagent was purchased from MERCK (Darmstadt, Germany). Dithiothreitol (DTT) and iodoacetamide (IAA) were purchased from GE Healthcare (Pittsburgh, PA). Trichloroethylene, triethylammonium bicarbonate (TEAB), trifluoroacetic acid (TFA), trypsin, formic acid, acetonitrile, isopropyl alcohol, pyrroline, and ammonium hydroxide solution were from Sigma-Aldrich (Shanghai, China).

### Depletion of high abundance serum proteins and sample clean up

Human sera was collected according to approved methods of the Ethics Committee of Shenzhen Second People’s Hospital. Samples (N = 10 each) from HCMV-infected, healthy controls and patients with hepatitis B virus infection were aliquoted and stored at −80 °C before use. Albumin and IgG were removed from serum using a ProteoExtract kit. Sample cleanup was conducted using a TCA-acetone protein precipitation procedure. First, 100% TCA was added to each sample to a final volume of 33%, and samples were vortexed for 1 min and incubated on ice for 3 h. Samples were centrifuged at 10,000 × g, 4 °C for 10 min. Supernatant was removed and ice cold 100% acetone was added. Samples were centrifuged at 10,000 × g, 4 °C for 5 min, and this was repeated twice. Acetone was removed and protein was re-dissolved in extraction buffer (6 M urea, 10 mM DTT). Quantification of the total proteins was performed following the manufacturer’s instructions for the 2-D Quant Kit (GE Healthcare, USA).

### Protein digestion, iTRAQ labeling and sample desalting

Samples were pooled for each group and 200 μg protein was fltered using an Amicon Ultra 3 K filter device (Millipore, location) for desalting and buffer exchange. Extraction buffer was replaced with 10 mM DTT in 100 mM TEAB and samples were reduced at 50 °C for 30 min. After the samples were cooled buffer was replaced with 20 mM IAA in 100 mM TEAB via 3 K filters. Samples were alkylated in the dark for 1 h and buffer was replaced with 100 mM TEAB via 3 K filters and 200 μg of trypsin was added to each sample and incubated overnight at 37 °C.

Digestion products were lyophilized and re-suspended in 20 μL 500 mM TEAB. iTRAQ reagents (113, 114 were used to label two replicates of pooled HCMV infected samples, 115, 116 were used to label two replicates of pooled healthy control samples) were prepared according to the manufacturer instructions: iTRAQ reagents were mixed with respective samples within and between biological replicates and incubated at room temperature for 2 h. Reactions were quenched by adding 100 μL water to each sample and incubating them for 30 min at room temperature. Labeled samples were pooled and lyophilized again, then re-dissolved in sample buffer (5% acetonitrile, 0.1% TFA). Desalting of peptide samples suing C18 spin columns was performed according to the manufacturer instructions, and samples were dried using a vacuum concentration system.

### Peptide separation by liquid chromatography and data acquisition by mass spectrometry

Samples were re-dissolved in 2% acetonitrile, 0.1% formic acid, and loaded on a ChromXP C18 (3 μm, 120 Å) nanoLC trap column. Online trapping and desalting was carried out at 2.0 μL/min for 10 min with 100% solvent A. Solvents were composed of water/acetonitrile/formic acid (A, 98/2/0.1%; B, 2/98/0.1%). Then, an elution gradient of 4–55% acetonitrile (0.1% formic acid) in a 90 min gradient was used on an analytical column (3 µm, 100 Å, 75 µm i.d. × 15 cm, Acclaim PepMap100, C18, Dionex). MS/MS analysis was performed with a TripleTOF 5600 System (AB SCIEX, Concord, ON) fitted with a Nanospray III source (AB SCIEX). Data were acquired using an ion spray voltage of 2.3 kV, curtain gas of 30 PSI, nebulizer gas of 5.5 PSI, and an interface heater temperature of 150 ^◦^C. The MS was operated with TOF-MS scans. For DIA, survey scans were acquired in 250 ms and as many as 30 product ion scans (80 ms) were collected if counts exceeded a threshold of 150 counts per second (counts/s) with a +2 to +5 charge-state. A rolling collision energy setting was applied to all precursor ions for collision-induced dissociation. Dynamic exclusion was set for ½ of peak width (~8 s).

### Protein identification, quantification and functional classification

MS/MS data were analyzed for protein identification and quantification using ProteinPilot Software v.4.5 (AB Sciex Inc.). The threshold of the local false discovery rate was estimated as 1.0% with the integrated PSPEP tool in the ProteinPilot Software, after searching against a decoy concatenated Uniprot human protein database (a total of 71434 entries) with MASCOT search engine. The search parameters were set as follows: iTRAQ quantification, cysteine modified with iodoacetamide, trypsin digestion, thorough searching mode and minimum protein threshold of 95% confidence (unused protein score >1.3). All peptide intensities were normalized to protein intensities by calculating the medians and then the data were arcsine transformed to obtain the data fitting a normal distribution. A student’s t-test was performed iteratively for each protein to test for statistical significances. Then, the data were transformed backwards to calculate the mean ± SD and 95% confidence intervals. The differential serum proteins were profiled by functional classification through Pather (http://www.pantherdb.org/).

### Protein validation using ELISA

Protein was quantified for each sample with a commercially available ELISA kit for human SAA1 and APOE (Abcam, Cambridge, MA) according to the manufacturer’s instructions. Briefly, equal amounts of crude sera from 15 HCMV patients, 15 healthy controls and 8 hepatitis B virus infected patients were measured using ELISA. Standards and samples were assayed in triplicate. Protein was calculated and analyzed using R 3.1.2.

### Protein validation with Western blot

Samples (N = 4 each) from HCMV-infected, healthy controls and hepatitis B virus infected patients were cleaned up using the TCA-acetone procedure. Samples were pooled with loading buffer and boiled at 96 °C for 7 min. After electrophoresis, proteins were transferred onto PVDF membranes and antibodies against GAPDH, SAA1 and APOE were diluted at 1:1000 in TBST buffer and incubated for 1 h at room temperature. Secondary goat anti-mouse antibody was diluted at 1:3000 in TBST and incubated for 1 h at room temperature. Protein bands were imaged using ECL substrate and an ImageQuant RT ECL System (GE Healthcare). Relative protein quantification was performed using ImageQuant TL-1D Analysis Tool. A student’s t-test was applied to test for statistical significance.

## Results

### Efficiency evaluation for IgG/albumin depletion

**Figure 1:**
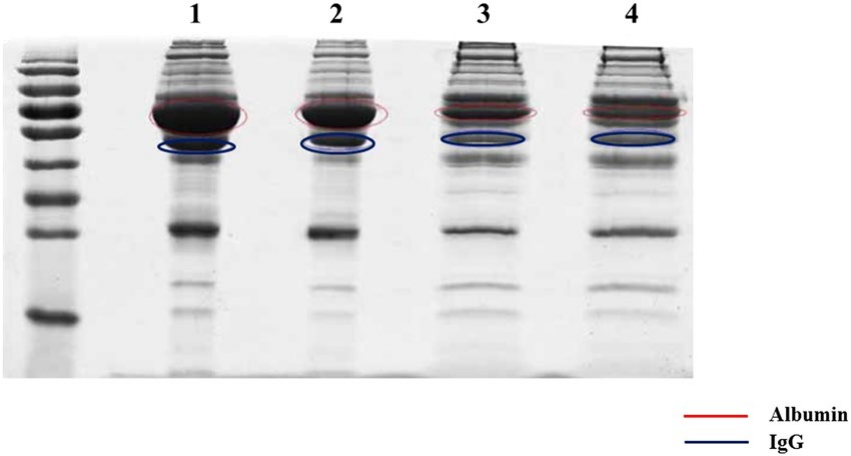
Removal of albumin and IgG from serum samples. 1 crude sera from healthy controls; 2 crude sera from HCMV-infected patients; 3 depleted sera from healthy control; 4 depleted sera from HCMV-infected patients.

A challenge for measuring serum protein with proteomic analysis is the high abundance of proteins that suppress signals of low abundance proteins during LC-MS/MS analysis. IgG and albumin are the two most abundant serum proteins, comprising 10–20% and 50–70% of total serum proteins, respectively. Thus to obtain data with high resolution and quality, a ProteoExtract kit was used to deplete serum albumin and IgG specifically. Densitometry analysis of stained protein bands showed that serum albumin and IgG were removed from the serum samples (Fig. 1).

### Quantitative analysis and functional classification of HCMV related differentially expressed proteins

**Figure 2:**
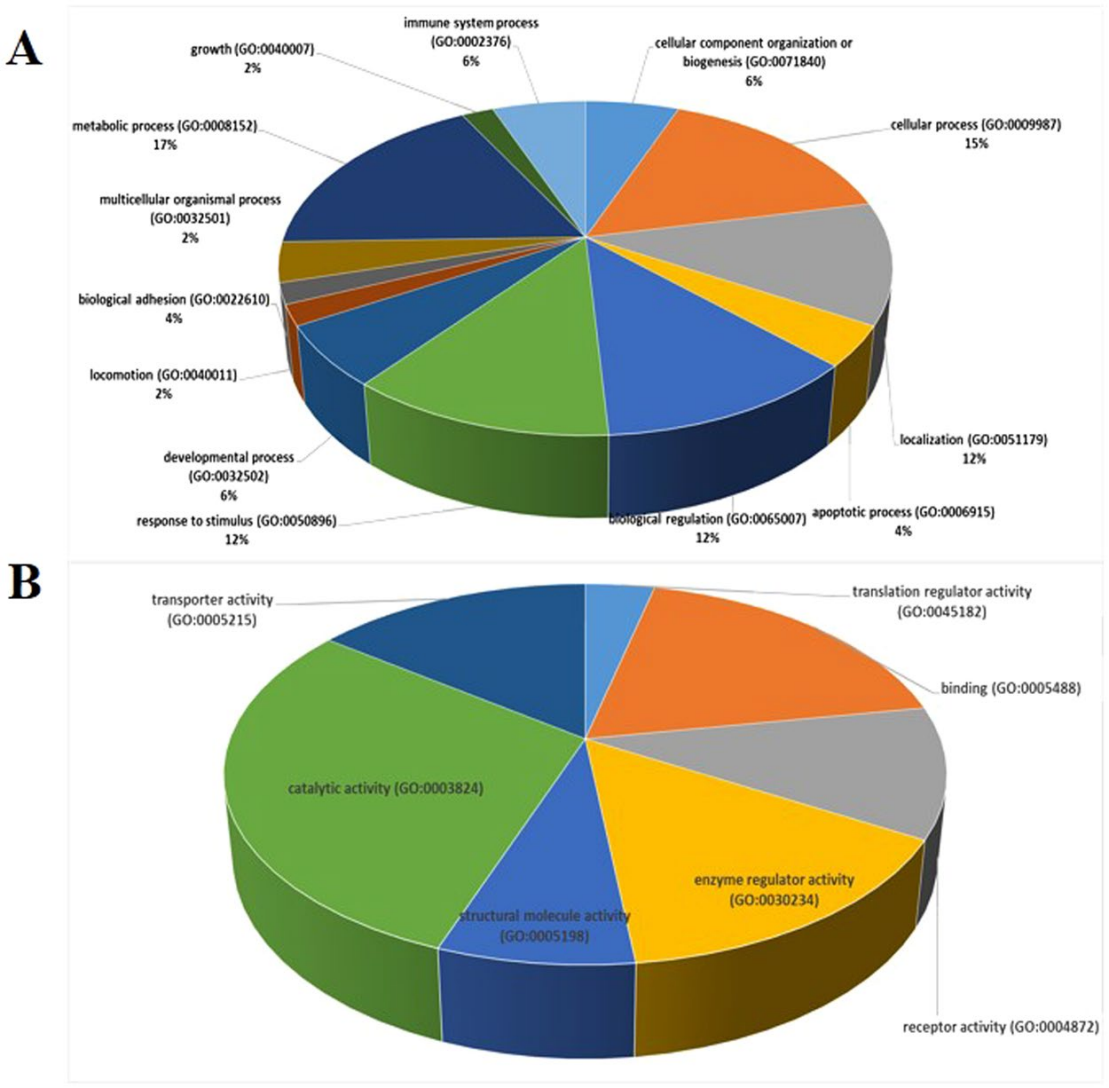
Functional protein classification using panther (http://www.pantherdb.org) with GO annotation data. Proteins were classified under the ontology of biological process and molecular function. (**A**) Protein classification based on biological processes. (**B**) Protein classification based on molecular function.

Before identification and quantification, peptides with less than 95% confidence or an unused protein score <1.3 were removed. Peptide intensities were transformed to protein signals by calculating medians and 538 proteins were identified in serum samples. Of these 21 were significantly (*P* < 0.05) altered in serum of HCMV-infected patients. Specifically, 5 proteins were up-regulated and 16 were down-regulated (Table 1). Differentially expressed serum proteins were classified by GO categories of biological processes and molecular functions (Fig. 2).

**Table 1:** Differential serum proteins in HCMV infected group.

Accession #	Protein Name	Ratio	Pval
sp|P0DJI8|SAA1_HUMAN	Serum amyloid A-1 protein	10.7647	0.0161
sp|P02741|CRP_HUMAN	C-reactive protein	6.0813	0.0154
sp|P01877|IGHA2_HUMAN	Ig alpha-2 chain C region	5.0119	0.0292
sp|Q9UK55|ZPI_HUMAN	Protein Z-dependent protease inhibitor	2.466	0.0551
sp|P04264|K2C1_HUMAN	Keratin, type II cytoskeletal 1	2.1478	0.0181
sp|P02748|CO9_HUMAN	Complement component C9	2.0701	0.0073
sp|P04196|HRG_HUMAN	Histidine-rich glycoprotein	0.4742	0.0041
sp|P01009|A1AT_HUMAN	Alpha-1-antitrypsin	0.4529	0.0133
sp|Q15582|BGH3_HUMAN	Transforming growth factor-beta-induced protein ig-h3	0.4406	0.0409
sp|P02749|APOH_HUMAN	Beta-2-glycoprotein 1	0.4325	0.0062
sp|P05155|IC1_HUMAN	Plasma protease C1 inhibitor	0.4169	0.014
sp|P11226|MBL2_HUMAN	Mannose-binding protein C	0.4093	0.0244
sp|P02656|APOC3_HUMAN	Apolipoprotein C-III	0.4055	0.0073
sp|P01008|ANT3_HUMAN	Antithrombin-III	0.3981	0.0197
sp|P10909|CLUS_HUMAN	Clusterin	0.3499	0.0278
sp|P02649|APOE_HUMAN	Apolipoprotein E	0.3281	0
sp|P01860|IGHG3_HUMAN	Ig gamma-3 chain C region	0.3251	0.0352
sp|O43866|CD5L_HUMAN	CD5 antigen-like	0.2884	0
sp|P01871|IGHM_HUMAN	Ig mu chain C region	0.263	0.0111
sp|Q9BXR6|FHR5_HUMAN	Complement factor H-related protein 5	0.2559	0.0545
sp|P01861|IGHG4_HUMAN	Ig gamma-4 chain C region	0.1406	0.0531
sp|P01880|IGHD_HUMAN	Ig delta chain C region	0.1318	0
sp|P01857|IGHG1_HUMAN	Ig gamma-1 chain C region	0.1148	0.0132
sp|P78417|GSTO1_HUMAN	Glutathione S-transferase omega-1	0.0112	0.0189

### APOE and SAA1 are potential biomarkers for identification of HCMV

**Figure 3:**
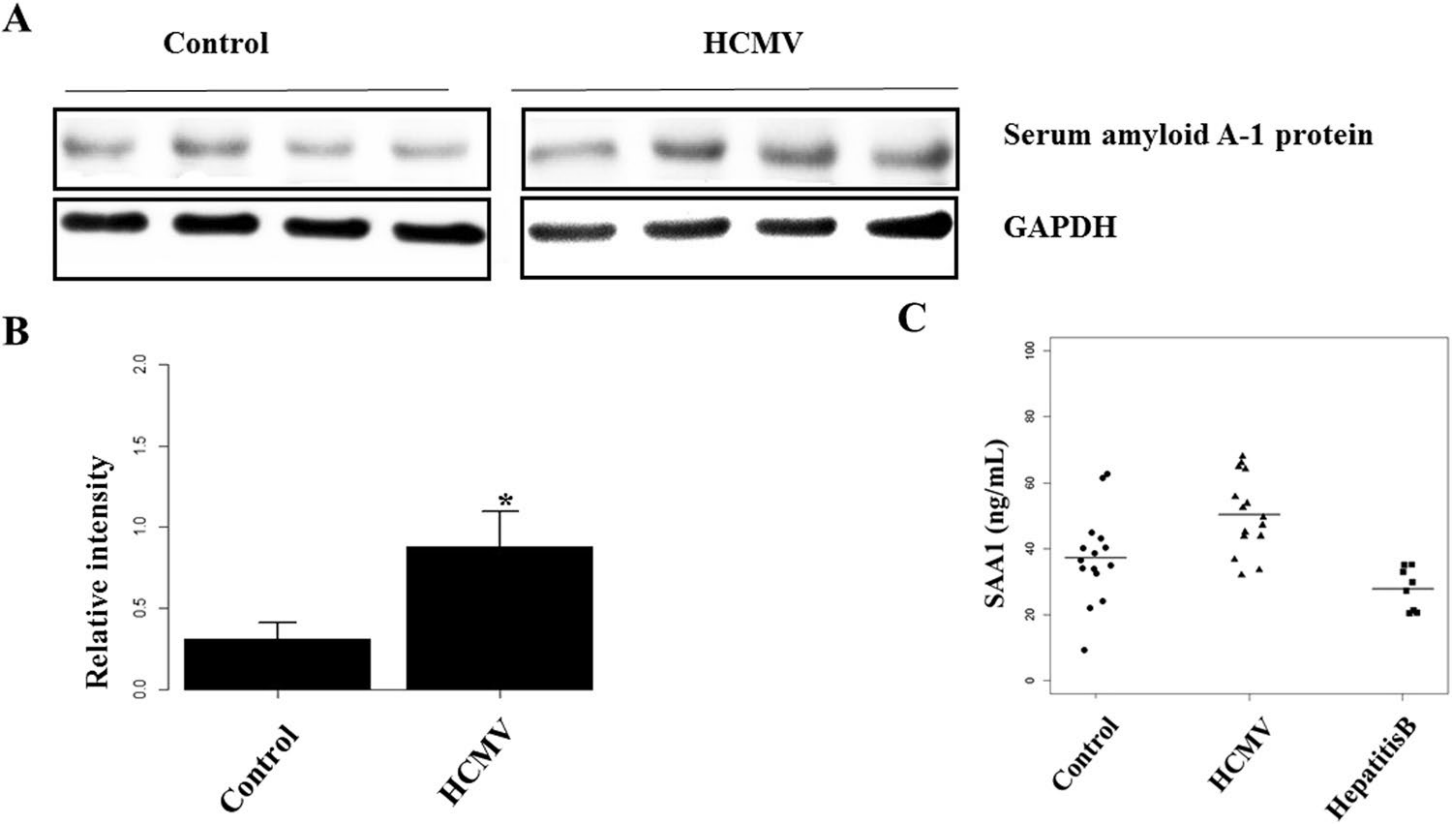
Validation data for potential serum markers for HCMV infection. Expression of proteins in crude sera of controls and infected patients validated by Western blot, and 15 more controls and patients by ELISA. An independent Student’s t-test was used for statistical validation. ***p < 0.001; (**A**) Protein bands of SAA1 in serum sample; (**B**) abnormal expression of SAA1 in sera from individuals with HCMV infection according to Western blot; (**C**) abnormal expression of SAA1 in sera from individuals with HCMV infection according to ELISA.

**Figure 4:**
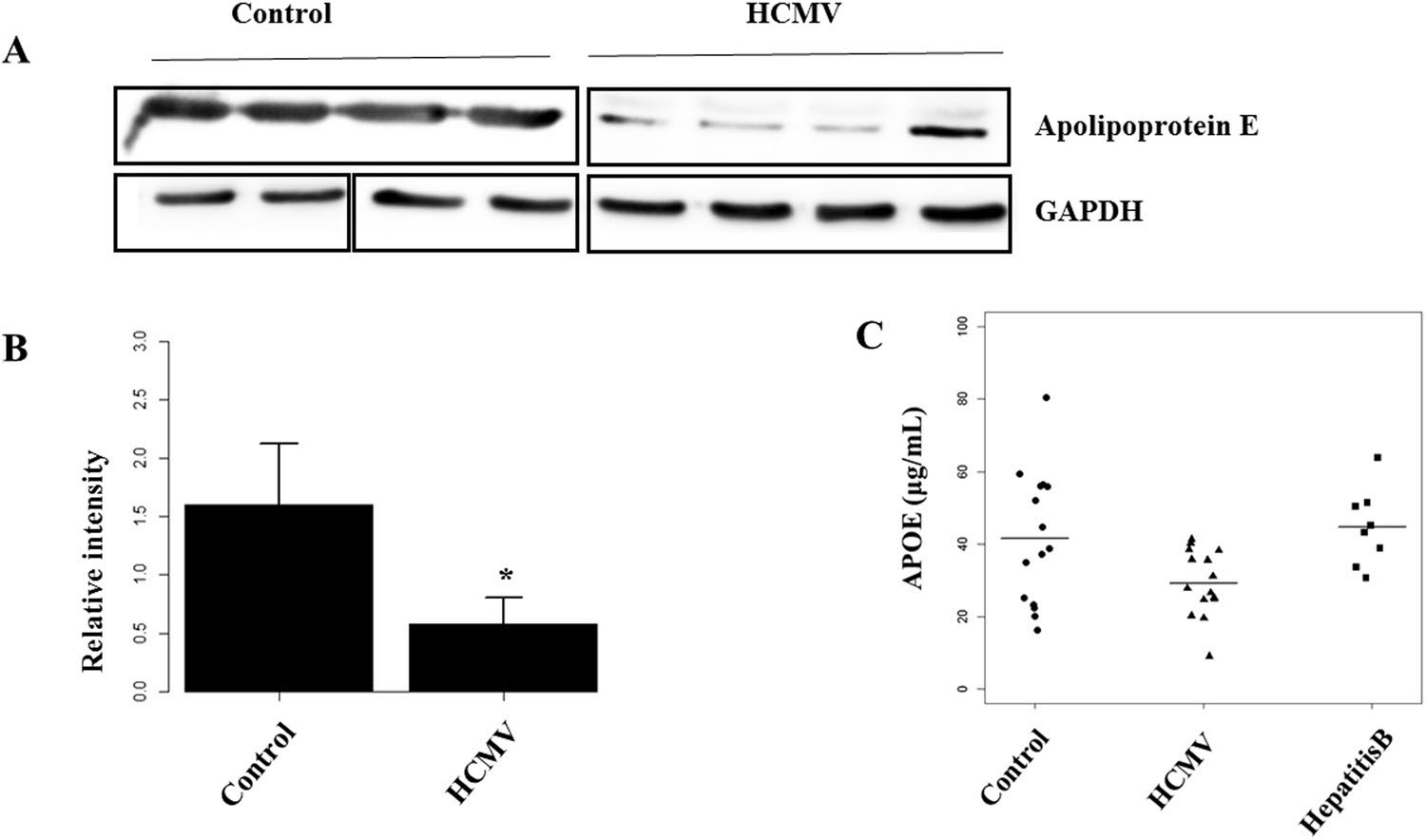
Validation data for potential serum markers for HCMV infection. Protein expression in crude sera of controls and patients enrolled in the proteomic analysis as validated by Western blot, and 15 more controls and patients as validated with ELISA. AN independent Student’s t-test was used for statistical validation. ***p < 0.001; (**A**) Protein bands of APOE in serum sample; (**B**) abnormal expression of APOE in sera from individuals with HCMV infection accoridn to Western blot; (**C**) abnormal expression of APOE in sera from individuals with HCMV infection according to ELISA.

**Figure 5:**
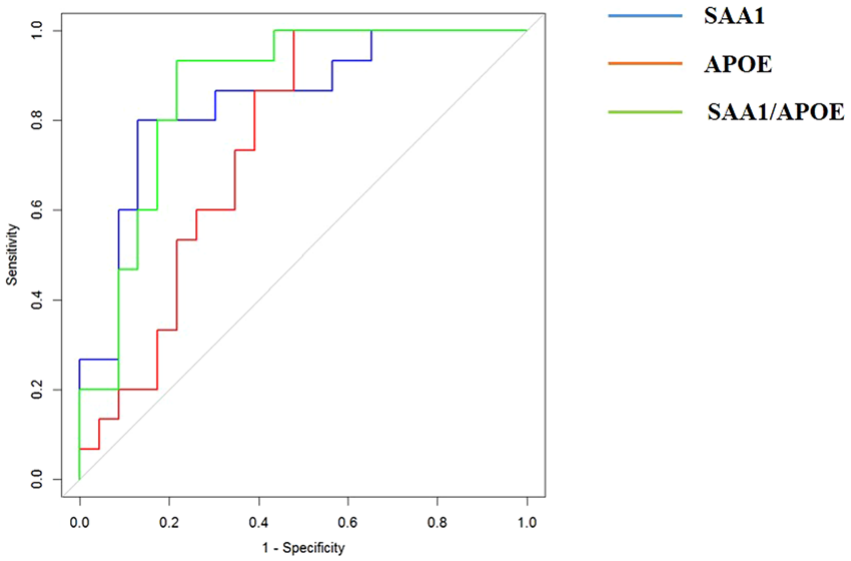
ELISA data based ROCcurves for SAA1 and APOE in discriminating HCMV infection from healthy controls. AUC for SAA1 and APOE were 0.78 and 0.67 respectively.

In all the differential serum proteins, SAA1 and APOE are not classic immune-related factors such as C-reactive protein, IgG, CD5 antigen-like protein *et al*. which may be altered in many other diseases. Moreover, SAA1 and APOE are with relatively high fold changes and low *P* values.. Western blot analysis indicated decreased APOE (Fig. 3AB) and increased SAA1 (Fig. 4AB) in HCMV-infected patients compared with healthy controls and hepatitis B virus infected patients respectively. ELISA confirmed down-regulation of APOE (Fig. 4C) and up-regulation of SAA1 (Fig. 3C) in HCMV-infected patients. To determine the potential ability of APOE and SAA1 to discriminate HCMV infections, healthy controls and hepatitis B virus infections, receiver operating characteristic (ROC) curves were generated based on ELISA data. The data of hepatitis B virus infections were mixed with healthy controls and results showed that SAA1 (AUC = 84.3%, specificity = 87.0%, sensitivity = 80.0%) was a better biomarker than APOE (AUC = 74.5%, specificity = 52.2%, sensitivity = 100.0%) in discriminating HCMV infections from healthy controls and hepatitis B virus infections (Fig. 5). However the co-occurrence (ratio between SAA1 and APOE) of the two proteins was with best performance (AUC = 86.7%, specificity = 78.3%, sensitivity = 93.3%) identifying HCMV infections.

## Discussion

Primary infection with HCMV is usually asymptomatic and can cause a lifelong latent infection of the host. Although in healthy carriers, no overt disease is observed, severe clinical symptoms are usually associated with HCMV reactivation in immunocompromised transplant patients and HIV sufferers. Transcription factors and histone proteins were found to influence HCMV gene expression profoundly and the virus-encountered cellular environment upon infection may have a critical role in determining a lytic or latent infection and subsequent reactivation from latency^9^. Research shows that the virus can interact with tumor suppressor p53 and induce excessive proliferation of smooth muscle cells which could eventually cause coronary restenosis after angioplasty^10^. HCMV infection is global and highly infectious. It is difficult to remove completely from the body, and the treatment is complicated and expensive. Thus, we need better early diagnostic methods for confirming HCMV infection.

To this end, we used a quantitative proteomic method to measure differential serum proteins and of the 538 proteins identified, 21 were differentially expressed. Among these differential proteins, 6 were up-regulated and 15 were down-regulated in HCMV-infected patients. Functional studies suggested enrichment of differential proteins mainly the in immune system and SAA1 and APOE were verified using Western blot and ELISA. ROC curve analysis was used to confirm protein accuracy as a biomarker.

SAA1 is a serum amyloid A family apolipoprotein and a major acute phase protein is highly expressed in response to inflammation and tissue injury. Different isoforms of SAA are expressed constitutively at different levels or in response to inflammatory stimuli. These proteins are produced predominantly by the liver. SAA1 was found to be protective for the development of amyloidosis in individuals with Familial Mediterranean fever^11^. SAA1 increases dramatically and then rapidly decreases. However, binding to high-density-lipoprotein can prolong the plasma half-life of SAA1^12^. SAA1 was reported to aggravate T-cell-mediated hepatitis by producing chemokine via activation of T-cells through the Toll-like receptor 2^13^.

APOE is a lipoprotein that combines with lipids to form complex molecules that are responsible for packaging cholesterol and other fats and carrying them through the bloodstream. Apolipoprotein E is a major component of low-density lipoproteins (VLDLs) which remove excess cholesterol from the blood and carry it to the liver for processing. Maintaining normal cholesterol is essential for prevention of disorders that affect the heart and blood vessels. IFN-gamma was found to increase lesion collagen content but reduce atherosclerotic lesion size, decrease lesion lipid accumulation and lesion cellularity in APOE knock-out mice^14^. Additional work indicated that deficiency of endogenous APOE could protect mice from angiotensin II-induced abdominal aortic aneurysm formation^15^. Our work not only gives insights into early diagnosis of HCMV but also provides a scientific basis for the future development of a rapid and specific serological diagnostic reagent.

### Ethics section

The collection of human sera was approved by the Ethics Committee of Shenzhen Second People’s Hospital.
